# Tripartite motif-containing protein 26 promotes colorectal cancer growth by inactivating p53

**DOI:** 10.1038/s41418-025-01463-1

**Published:** 2025-02-24

**Authors:** Zhihui Tan, Hyun Min Ko, Parnian Naji, Rong Zhu, Jieqiong Wang, Shibo Huang, Yiwei Zhang, Shelya X. Zeng, Hua Lu

**Affiliations:** 1 Department of Biochemistry & Molecular Biology and Tulane Cancer Center, Tulane University School of Medicine, New Orleans, LA 70112 USA; 2 Department of Gynecology, Xiang-Ya Hospital, Central South University, Changsha, 410008 China; 3 Department of Surgery, Division of Surgical Oncology, University Hospitals Cleveland Medical Center, Cleveland, OH 44106 USA; 4 School of Life Sciences, Hubei University, Wuhan, Hubei 430062 China; 5 The Research Center for Clinical Trials, The First Affiliated Hospital, Nanchang University, Nanchang, Jiangxi 330006 China

**Keywords:** Oncogenes, Tumour-suppressor proteins

## Abstract

Tripartite motif-containing protein 26 (TRIM26) is an E3 ubiquitin ligase that exhibits divergent roles in various cancer types (oncogenic and anti-oncogenic). This study investigates the interaction of TRIM26 with the tumor suppressor protein p53 in colorectal cancer (CRC) cells by performing a comprehensive set of biochemical, cell-based assays, and xenograft experiments. As a result, we found that overexpression of TRIM26 significantly enhances CRC cell proliferation and colony formation, while knockdown of TRIM26 suppresses these processes. Xenograft experiments further validated the tumor-promoting role of TRIM26 in CRC. Supporting this is that TRIM26 is highly expressed in human CRC tissues as revealed by our analysis of the TCGA database. Biochemically, TRIM26 directly bound to the C-terminus of p53 and facilitated its ubiquitination, resulting in proteolytic degradation and attenuated p53 activity independently of MDM2. Also, TRIM26 increased the MDM2-mediated ubiquitination of p53 by binding to MDM2’s C-terminus. This study uncovers the oncogenic potential of TRIM26 in CRC by inhibiting p53 function. Through its ubiquitin ligase activity, TRIM26 destabilizes p53, consequently promoting CRC cell proliferation and tumor growth. These findings shed light on the complex involvement of TRIM26 in cancer and identify this ubiquitin ligase as a potential therapeutic target for future development of CRC treatment.

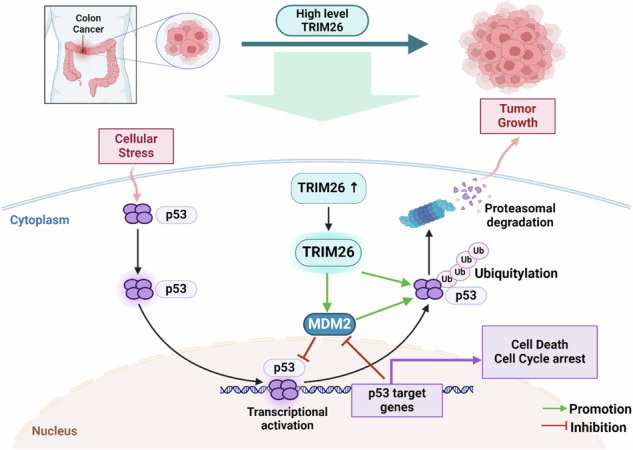

## Introduction

Tripartite motif-containing protein 26 (TRIM26) is one of the TRIM family members and is composed of the N-terminal RING, B-box 2, Coiled coil, and C-terminal PRY-SPRY domains [1]. Its gene is located and clustered with six other TRIM family members on chromosome 6p21.33-6p22.2 [2]. Although functioning as E3 ubiquitin ligases, TRIMs have been shown to play diverse roles in biological processes, such as cellular proliferation, cell cycle advancement, DNA maintenance, ferroptosis, and autophagy [3–7]. TRIM26’s functions in cancers and their underlying mechanisms just began to be unveiled less than a decade ago. On the one hand, TRIM26 was reported to play a potential tumor-suppressive role in some types of human cancers. For example, overexpression of TRIM26 inhibited the growth of non-small cell lung carcinoma cells by suppressing the PI3K/AKT signaling pathway [8] and also, led to the suppression of proliferation, metastasis, and growth of papillary thyroid cancer cells [9]. Similarly, TRIM26 could also act as a tumor suppressor of hepatocellular carcinoma (HCC) by promoting ferroptosis, and its downregulation was associated with poor prognosis of HCC [10, 11]. This result was later validated by another study showing that TRIM26 can suppress HCC growth and migration by ubiquitinating and degrading Zinc-finger E-box-binding homebox1 (ZEB1) [12], an oncogenic transcriptional factor crucial for HCC growth and metastasis [12–14]. On the other hand, more recent studies showed that TRIM26 surprisingly plays an oncogenic role in different types of tumors. Knockdown of TRIM26 led to the inhibition of proliferation, migration, and invasion of bladder cancer cells by impeding the AKT/ GSK3β/β-catenin pathway [4]. Also, TRIM26 was found to stabilize SOX2 protein and enhance its oncogenic activity in glioblastoma via its C-terminal PRYSPRY domain without engaging its Ring domain and E3 ligase activity [15]. Silencing TRIM26 in nasopharyngeal carcinoma is another oncogenic role of TRIM26 that attenuates the immune response mediates by downregulating the Natural killer cells’ activity [16]. These studies suggest that TRIM26 appears to play both oncogenic and anti-oncogenic roles, depending on the types of tissues, cells, or tumors. However, no study has explored whether these roles of TRIM26 might be dependent or independent of p53 or not, which is the most important genome guardian as a tumor suppressor.

The tumor suppressor p53 can activate the expression of various genes crucial for cell-cycle control, senescence, DNA repair, apoptosis, necrosis, autophagy, and ferroptosis in response to various stressors [17]. p53 protein is composed of an N-terminal transactivational domain (TAD), a central DNA binding domain (DBD), a tetramerization domain, and a C-terminal regulatory region [17] and functions as a homotetrameric transcription factor [18, 19]. MDM2 regulates the p53 stability via ubiquitination-dependent proteolysis in a negative feedback fashion [20–22]. In addition to MDM2, p53 can be ubiquitinated by other ubiquitin ligases under different cellular and pathological conditions [23–25]. In our search for possible new p53 regulators, we identified TRIM26 as a new candidate that is a ring E3 ligase targeting p53 ubiquitination. This resulted in proteolytic degradation, consequently inactivating p53 and promoting the growth and proliferation of colorectal cancer cells in culture and xenograft. Additionally, it directly interacts with both p53 and MDM2, thereby further enhancing p53 ubiquitination facilitated by MDM2. TRIM26 is highly expressed in colorectal adenocarcinomas as well as in other types of cancers as revealed by our analysis of the TCGA database. Thus, our results as further described below demonstrate that TRIM26 acts as an oncoprotein to promote colorectal cancer growth by inactivating p53.

## Results

### TRIM26 promotes the proliferation and growth of colorectal cancer cells

**Fig. 1:**
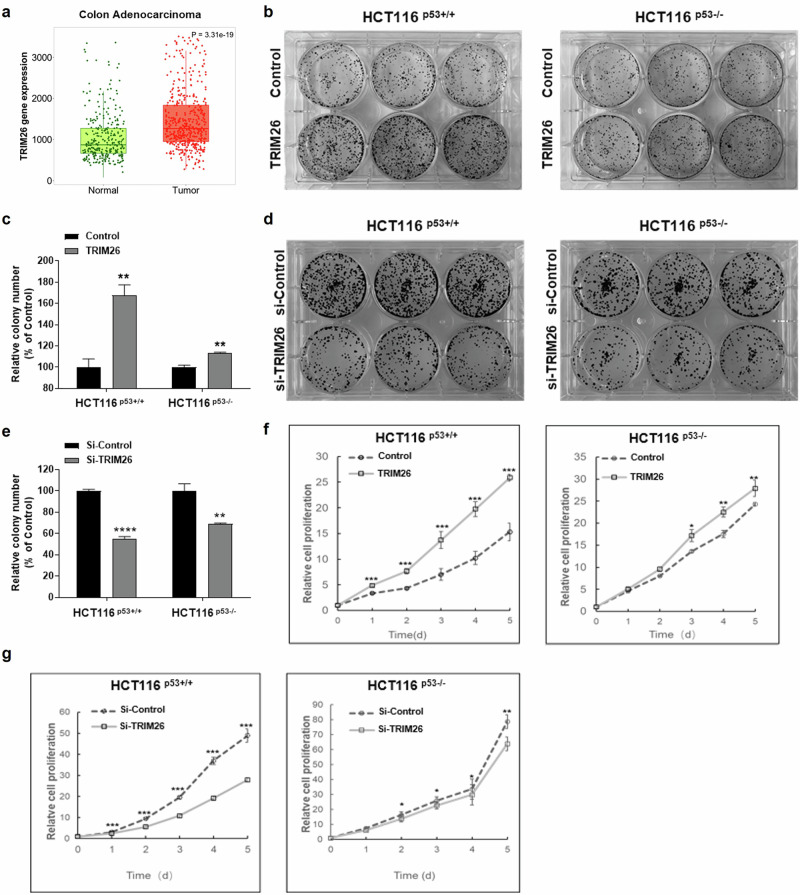
TRIM26 is overexpressed in colon adenocarcinoma and promotes cell growth and proliferation. **a** TRIM26 mRNA expression was determined using RNA-sequencing in normal colon tissues (*n* = 315) and colon cancer (*n* = 469) from TNMplot.com [40]. **b–e** TRIM26 promotes colony formation. The cells were transfected with Flag-TRIM26 or Si-TRIM26 in HCT116^(p53+/+)^ and HCT116^(p53–/–)^ and seeded in 6-well plates for 14 days: (**b**): Cell colony formation after overexpression of TRIM26. (**d**): Cell colony formation after knockdown of TRIM26. Histograms indicate the relative colony number (**c**): overexpression of TRIM26, (**e**): knockdown of TRIM26. The Student’s two-tailed t test was used to present mean differences among groups. Data are mean ± s.e.m. **P < 0.01, ****P  <  0.0001 vs. control. TRIM26 promotes cell proliferation, partially in a p53-dependent manner in colon cancer cells. Cell proliferation after (**f**) overexpression and (**g**) knockdown of TRIM26 in HCT116^(p53+/+)^ and HCT116^(p53–/–)^; The cells were transfected with Flag-TRIM26 or Si-TRIM26 for 24 h and then split into 96-well plates, CCK-8 was added for 1 h and OD-450 was measured each day and for 5 days. The statistics analyses were performed by Student’s two-tailed t test. Data are represented as mean ± s.e.m. *P < 0.05, **P < 0.01, ***P < 0.001 vs. control.

**Fig. 2:**
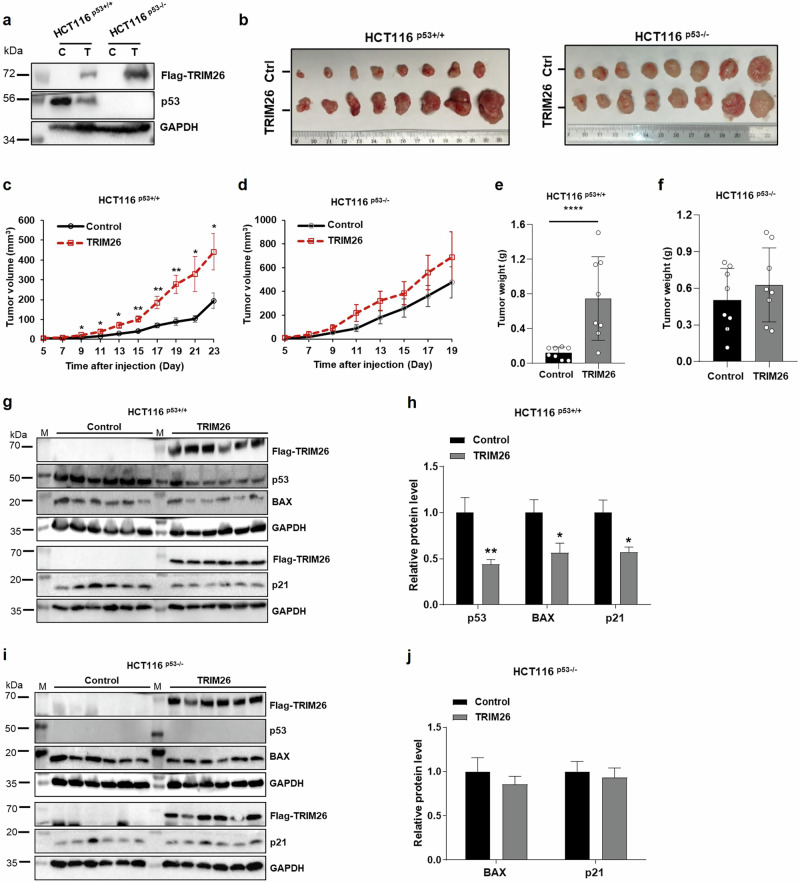
TRIM26 overexpression induced tumor progression in xenograft tumor models in a p53-dependent way. Stable cell lines were constructed in colon cancer cells HCT116^(p53+/+)^ and HCT116^(p53–/–)^ using PLVX-flag-TRIM26 as positive and PLVX-vector as control. Stable cells were injected into the Nude mice (8 mice in each group). The tumor volumes were measured 5 days after the cell injection and tumor weights were measured for 23 days in HCT116^(p53+/+)^ group and for 21 days in HCT116^(p53-/-)^ group. **a** The overexpressed TRIM26 protein level was confirmed by WB analysis immediately following implantation of TRIM26-overexpressed HCT116 stable cells. **b** Photographs of the xenograft tumors retrieved. **c**, **d** The average tumor volumes at the indicated time are depicted. **e**, **f** tumor weights were measured immediately after isolation. The statistics analyses were performed by Student’s two-tailed t test. The data are plotted as mean ± s.e.m. (n = 8/group); *P < 0.05, **P < 0.01, ****P  <  0.0001 vs. control. **g–j** The protein levels of p53, BAX, p21 were measured by WB analysis after tumor tissues were isolated. **g**, **h** The protein levels were measured in 6 mice injected with stable overexpressed TRIM26 or control HCT116^(p53+/+)^ cells, histograms indicate the relative average expression level. **i**, **j** The protein levels were measured in 6 mice injected with stable overexpressed TRIM26 or control HCT116^(p53-/-)^ cells, histograms indicate the relative average expression level. The statistics analyses were performed by Student’s two-tailed t test. The results were plotted as mean ± s.e.m. (n = 6/group); *P < 0.05, **P < 0.01 vs. control.

Since TRIM26 has been shown to play both oncogenic and anti-oncogenic roles pending on types of cells and cancers [4, 9], we wondered if it might affect the growth of colorectal cancer (CRC) cells once we identified this protein as a potential p53-binding protein as described later in this report. Our TCGA database analysis of the TRIM26 gene expression as shown in RNA-sequencing data from TNMplot.com (Fig. 1a) showed that its RNA level is significantly higher in colon adenocarcinoma tissues than that in healthy colon tissues. This result suggested that TRIM26 might be favorable to the development of this cancer. The same pattern was found in a variety of tissue types of cancer, including bladder cancer and skin melanoma (S Fig. 1). To test if TRIM26 might play an oncogenic role in cancer cells, this study focused on CRC cells. To do so, we performed a set of colony formation and survival assays in HCT116 CRC cells (see “Materials and Methods”). As shown in Fig. 1b–e, overexpression of TRIM26 promoted (Fig. 1b, c), but knockdown of TRIM26 suppressed (Fig. 1d, e), colony formation of CRC cells. Notably, this effect was much more pronounced in p53-proficient HCT116 cells than in p53-null HCT116 cells. As part of result validation, p53-containing SK-Mel-5 and SK-Mel-147 cell lines, derived from skin melanoma, underwent testing, yielding outcomes consistent with those obtained from the HCT cell line (S Fig. 2a–d). Consistent with these results, overexpression of TRIM26 also enhanced (Fig. 1f), while knockdown of TRIM26 inhibited (Fig. 1g), the proliferation of CRC cells. Again, this effect appeared to be in part dependent of p53, as it was more significant in HCT116^p53+/+^ than HCT116^p53-/-^ cells when TRIM26 was knocked down (Fig. 1f, g). These results suggest that TRIM26 can promote the proliferation and growth of CRC and melanoma cells in part dependently of p53, though it might also possess a p53-independent oncogenic activity in the cancer cells.

### TRIM26 promotes growth of xenograft colorectal tumors by suppressing p53 activity

To further validate the potential oncogenic role of TRIM26 as suggested in our cell-based assays above (Fig. 1 and S Figs. 1–2), we performed a xenograft experiment by inoculating HCT116^p53+/+^ or HCT116^p53–/–^ cells that harbored ectopically expressed TRIM26 (Fig. 2a). The growth of HCT116 cells-derived xenograft tumors was monitored for 3 weeks or so and then harvested for further analyses on day 23 or day 21. As shown in Fig. 2, overexpression of TRIM26 (Fig. 2b) in the CRC cells markedly promoted the growth of the xenograft tumors. This promotion was more pronounced in HCT116^p53+/+^ cells-derived tumors than in HCT116^p53–/–^ cells-derived tumors in terms of tumor volumes (compare 2c to 2d) and tumor weights (compare 2e to 2f). This result indicated that TRIM26 might promote tumor growth by suppressing p53 activity. Indeed, this was the case as ectopic TRIM26 led to the reduction of p53 protein levels in HCT116^p53+/+^ cells-derived tumors (Fig. 2g, h), but not in HCT116^p53-/-^ cells-derived tumors (Fig. 2i, j). Consistently, protein levels of p53 target genes, such as BAX and p21, were also markedly reduced in HCT116^p53+/+^ cells-derived tumors (Fig. 2h), but not in HCT116^p53-/-^ cells-derived tumors (Fig. 2j). Taken together, these results indicate that TRIM26 can promote the growth of human CRC cells-derived xenograft tumors likely by suppressing p53 activity.

### TRIM26 reduces p53 level and activity in colorectal cancer cells

**Fig. 3:**
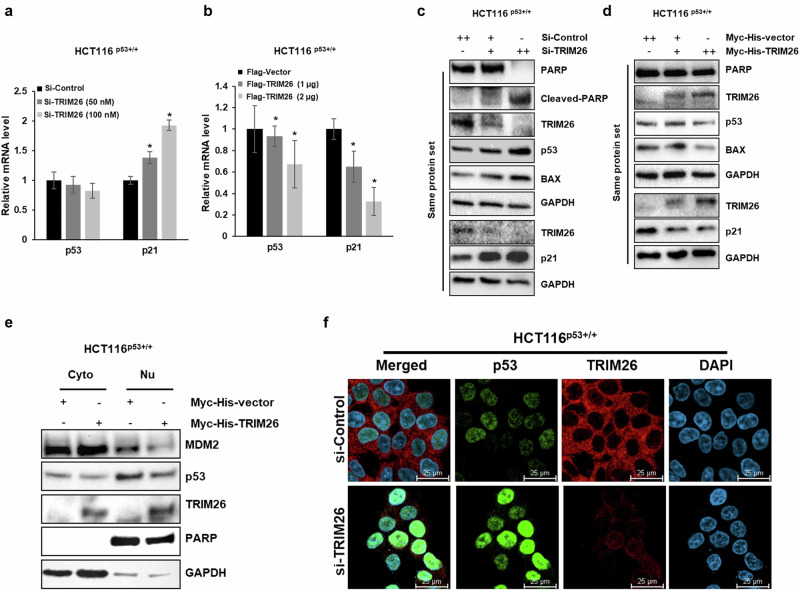
TRIM26 inhibits wt p53 in protein level. **a**, **b** p53 and p21 mRNA levels measured by Q-PCR in HCT116^(p53+/+)^ cells. The cells were transfected with Si-TRIM26, Si-control, Flag-TRIM26 or Flag-control for 48 h and harvested for Q-PCR test with a specific primer. Statistical analyses were performed using the student’s two-tailed *t* test. Data are represented as mean ± s.e.m. *P < 0.05. **c**, **d** Protein levels after (**c**) knocking down or (**d**) overexpression TRIM26 in HCT116^(p53+/+)^ cells. The cells were transfected with Si-TRIM26, Si-control, Flag-TRIM26 or Flag-control for 48 h and harvested for WB analysis with indicated antibodies. **e** The cell fractionation assay was conducted after cells were transfected with Myc-His-TRIM26 or Myc-His-control for 48 h. Cyto cytoplasm, Nu nucleus. **f** IF staining shows induction of p53 in nucleus by depletion of TRIM26. Cells are transfected with TRIM26 siRNA for 48 h. Afterwards, cells are fluorescently stained with anti-TRIM26 and anti-p53. DAPI was used as an indicator of nuclear. (Scale bar, 25 μm).

**Fig. 4:**
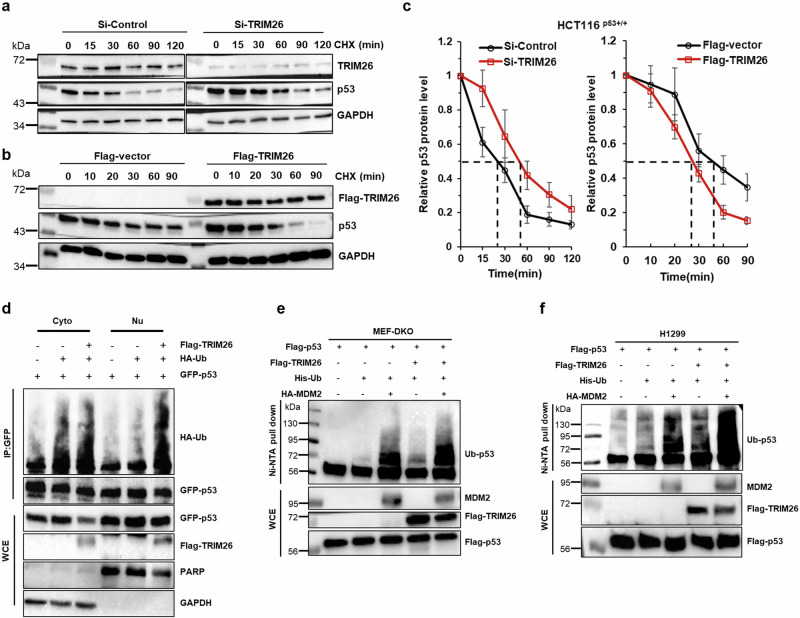
TRIM26 ubiquitinates p53 and also enhances MDM2 ubiquitination of p53. **a**, **b** Half-life of p53 in TRIM26-knockdown and TRIM26-overexpressed HCT116^(p53+/+)^ cells. WB detected the protein levels of p53. The HCT116^(p53+/+)^ cells were transfected with Si-TRIM26, Si-control, Flag-TRIM26, or Flag-control for 48 h, and then treated with 100 µg/ml of CHX and harvested at different time points after the treatment for WB analysis with indicated antibodies. An equal amount of proteins (50 µg) was loaded in each lane. **c** p53-half-lives were quantified by densitometry and plotted against time. **d** Ubiquitination of exogenous p53 in HCT116^(p53-/-)^ cells was explored with or without TRIM26 overexpression. Cells were transfected with plasmids indicated in the figure for 48 h and treated with 20 uM of MG132 for 6 h before being collected. Proteins were fractionated and then pulled down using GFP antibody followed by WB analysis with indicated antibodies. **e**, **f** Ubiquitination assays of exogenous wt p53 were conducted in (**e**) MEF-DKO cells and (**f**) H1299 cells with or without TRIM26 overexpression. Cells were transfected with plasmids indicated in the figure for 48 h and treated with 20 uM of MG132 for 6 h before being collected. Proteins were extracted for Ni-NTA beads pulldown assays followed by WB analysis with indicated antibodies. Ubiquitinated proteins and total proteins were detected by WB analysis with indicated antibodies. An equal amount of proteins (50 µg) was loaded in each lane.

To understand how TRIM26 might regulate p53 level and activity, we conducted a set of cell-based assays by either knocking down or overexpressing TRIM26 in HCT116^p53+/+^ cells. Knockdown of TRIM26 by its specific siRNA induced (Fig. 3a, c), but overexpression of TRIM26 reduced (Fig. 3b, d), the protein and RNA levels of p21 in a dose-dependent fashion. Interestingly, knockdown of TRIM26 by its specific siRNA induced (Fig. 3c), while overexpression of TRIM26 reduced (Fig. 3d) the protein level of endogenous p53 in these CRC cells. However, neither approach significantly affected the RNA levels of p53 (Fig. 3a, b). Correspondingly, knockdown of TRIM26 led to an increase in BAX and cleaved PARP levels (Fig. 3c), while overexpression of TRIM26 reduced their expression in the cells (Fig. 3d). These findings suggest that TRIM26 may suppress p53-dependent apoptosis (S Fig. 3a, b). Along with these findings, we conducted tests on SK-Mel-5 and SK-Mel-147 cell lines to verify the impact of TRIM26 on p53-dependent apoptosis (S Fig. 4a, b). These results not only were consistent with the xenograft results as shown in Fig. 2g, i, but also suggested that TRIM26 might regulate the protein, but not RNA, level of p53. Furthermore, we conducted cell fractionation assay (Fig. 3e) and immunofluorescence (IF) analysis (Fig. 3f) to determine the intracellular localization of p53 protein influenced by TRIM26. Our findings revealed that overexpression of TRIM26 reduced the nuclear p53 protein levels (Fig. 3e), while knocking down TRIM26 resulted in the increase of nuclear p53 levels (Fig. 3f ).

To test if TRIM26 might affect p53 stability, we assessed the half-life of the p53 protein by either knocking down or overexpressing TRIM26 in HCT116 cells. As shown in Fig. 4a–c, knockdown of TRIM26 extended the half-life of p53, whereas overexpression of TRIM26 shortened p53’s half-life. These results indicate that TRIM26 can destabilize p53 protein, consequently inhibiting its activity.

### TRIM26 ubiquitinates p53 and also enhances MDM2 ubiquitination of p53

**Fig. 5:**
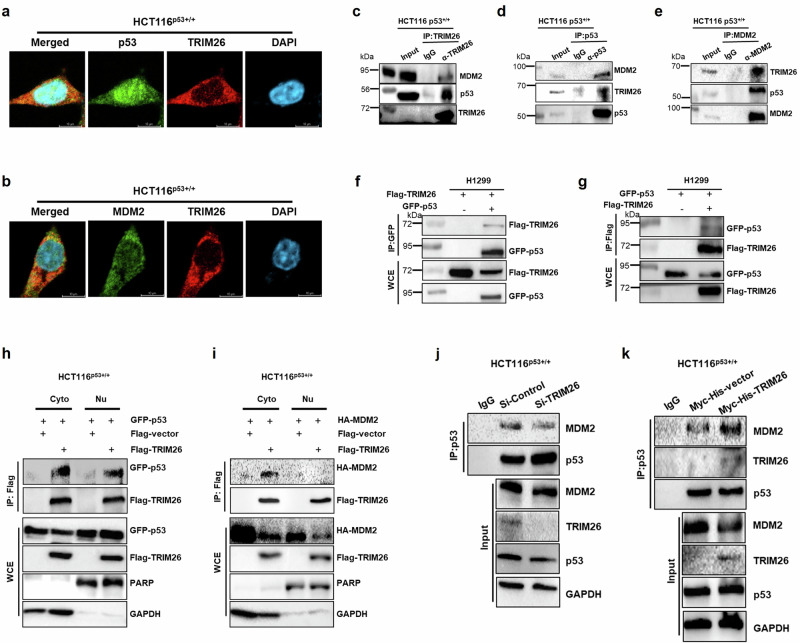
TRIM26 interacts with both p53 and MDM2 and boosts their interaction. **a**, **b** co-localization of endogenous TRIM26, p53 and MDM2 in CRC cells. **a** p53, and (**b**) MDM2 with TRIM26 were detected in HCT116^(p53+/+)^ cells by using IF analysis. Following cell fixation, the cells underwent permeabilization, blocking, and staining with primary antibodies as indicated, then followed by secondary antibody staining. DAPI was used to indicate the nucleus. (Scale bar, 10 μm). **c–e** Co-IP-WB analysis shows that the interaction between endogenous TRIM26 and p53 and MDM2 is detected in HCT116^(p53+/+)^ cells by pulling down the respective (**c**) TRIM26 or (**d**) p53 or (**e**) MDM2 antibodies. **f** H1299 cells were transfected with Flag-TRIM26, with or without GFP-p53, for 48 h. GFP beads were used to pull down interacting proteins, followed by WB analysis with the indicated antibodies. **g** H1299 cells were transfected with GFP-p53, with or without Flag-TRIM26, for 48 h. Flag beads were used to pull down interacting proteins, followed by WB analysis with the indicated antibodies. HCT116^(p53+/+)^ cells were transfected with (**h**) GFP-p53 or (**I**) HA-MDM2 along with either Flag-vector or Flag-TRIM26 for 48 h, followed by cell fractionation. The fractionated samples were then subjected to pull-down using Flag beads to isolate interacting proteins, and Western blot analysis was performed using the indicated antibodies. **j** Depletion of TRIM26 inhibits the endogenous p53-MDM2 complex. Si-TRIM26 or Si-control was transfected into HCT116 ^(p53+/+)^ cells and then treated with MG132 (20 μM) for 6 h prior to harvest. Harvested samples were analyzed by co-IP-WB using anti-p53 antibody. **k** Overexpression of TRIM26 enhances endogenous p53-MDM2 complexes. Myc-His-TRIM26 or Myc-His-control was transfected into HCT116 ^(p53+/+)^ cells and then treated with MG132 (20 μM) for 6 h prior to harvest. Harvested samples were analyzed by co-IP-WB using an anti-p53 antibody.

Since TRIM26 possesses an intrinsic E3 ubiquitin ligase activity [8], Next, we performed a cellular p53 ubiquitination assay using cell fraction samples containing ectopic TRIM26 and p53 to determine whether TRIM26 can mediate p53 degradation through ubiquitination (Fig. 4d). Interestingly, we found that TRIM26 mainly ubiquitinate p53 in the nucleus. In addition, we performed a cell-based p53 ubiquitination assay with MDM2 as a positive control in p53/MDM2 double knockout murine embryonic fibroblast (MEF-DKO) cells. As shown in Fig. 4e, overexpression of TRIM26 led to a basal level of p53 ubiquitination independently of MDM2. Although its ubiquitination activity toward p53 was not as strong as MDM2’s activity, TRIM26 could further enhance p53 ubiquitination in the presence of MDM2 (last lane of Fig. 4e and S Fig. 5a). These results were repeated in human lung cancer H1299 cells that are p53-deficient (Fig. 4f and S Fig. 5b). Taken together, these results indicate that TRIM26 can mediate p53 proteolytic degradation by ubiquitinating it and boost MDM2-mediated p53 ubiquitination.

### TRIM26 interacts with both p53 and MDM2 and also boosts their interaction

**Fig. 6:**
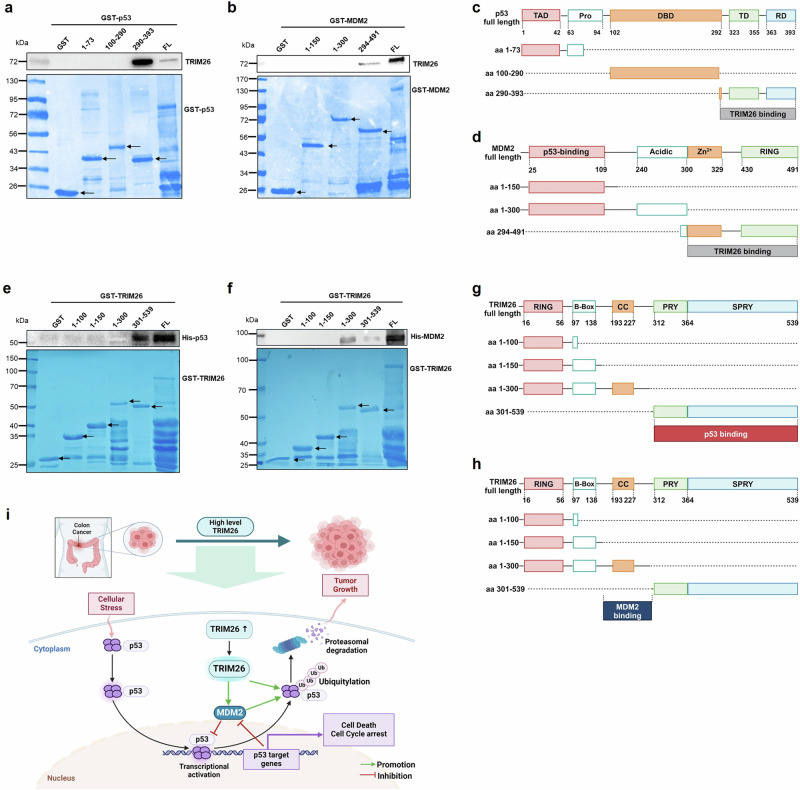
TRIM26 interacts with p53 and MDM2 via C-termini. **a** Purified TRIM26 was pulled down by GST-p53 fragments. Flag-TRIM26 was digested by PreScission Protease and incubated with each of the GST-p53 fragments in beads at RT for 40 min. Upper panel: Bound TRIM26 was detected by WB analysis with the TRIM26 antibody; lower panel: GST-p53 fragments were stained by Coomassie blue staining. Black arrows indicate GST-p53 fragments bands. **b** Purified TRIM26 was pulled down by GST-MDM2 fragments. TRIM26 was digested by PreScission Protease and incubated with each of the GST-MDM2 fragments in beads at RT for 40 min. Upper: WB analysis of TRIM26 as detected by the TRIM26 antibody; Lower: GST-MDM2 fragments were stained by Coomassie blue staining. Black arrows indicate GST-MDM2 fragments bands. An equal amount of proteins (50 µg) was loaded in each lane. **c** Schematic representation of TRIM26 binding domain of p53. **d** Schematic representation of TRIM26 binding domain of MDM2. **e** Purified p53 was pulled down by GST-TRIM26 fragments. His-p53 was incubated with each of the GST-TRIM26 fragments in beads at RT for 40 min. Upper panel: Bound His-p53 was detected by WB analysis with the His antibody; lower panel: GST-TRIM26 fragments were stained by Coomassie blue staining. Black arrows indicate GST-TRIM26 fragments bands. **f** Purified MDM2 was pulled down by GST-TRIM26 fragments. His-MDM2 was incubated with each of the GST-TRIM26 fragments in beads at RT for 40 min. Upper panel: Bound His-MDM2 was detected by WB analysis with the His antibody; lower panel: GST-TRIM26 fragments were stained by Coomassie blue staining. Black arrows indicate GST-TRIM26 fragments bands. **g** Schematic representation of p53 binding domain of TRIM26. **h** Schematic representation of MDM2 binding domain of TRIM26. **i** A schematic showing that TRIM26 promotes CRC survival and growth by inactivating p53 via two mechanisms: (1) directly binding to p53 and ubiquitinating it (2) and boosting MDM2 ubiquitination of p53 by directly binding to both the proteins. As a result, TRIM26 promotes the proliferation of colon cancer cells and tumor formation.

To better understand the interactions between TRIM26, p53, and MDM2, we first conducted an immunofluorescence (IF) analysis to examine the localization of endogenous TRIM26, p53, and MDM2 (Fig. 5a, b). Interestingly, we found that TRIM26 is primarily distributed in the cytoplasm, while it colocalizes predominantly with p53 in the nucleus and with MDM2 in the cytoplasm. To further support the physical association of TRIM26 with p53 and MDM2 in vivo at the exogenous level, we investigated the localization of these proteins by overexpressing TRIM26, p53, and MDM2 individually or together (S Fig. 6a–f). Intriguingly, when p53 was overexpressed alone, it was evenly distributed between the cytoplasm and nucleus (S Fig. 6b). However, when co-overexpressed with TRIM26, p53 predominantly localized to the nucleus and colocalized with TRIM26 (S Fig. 6d, e). Similarly, MDM2, which showed a predominant nuclear localization when overexpressed alone (S Fig. 6c), was found to translocate from the nucleus to the cytoplasm upon co-overexpression with TRIM26, resulting in colocalization with TRIM26 in the cytoplasm (S Fig. 6f). Furthermore, to determine whether TRIM26 regulates p53 stability through directly binding to p53 and MDM2, we conducted a series of in vitro and cellular protein-protein interaction assays.

First, we performed pull-down assays using TRIM26, p53, and MDM2 antibodies and confirmed in Fig. 5c–e that these proteins bind to each other, forming a complex at endogenous levels. We also conducted reciprocal co-immunoprecipitation (co-IP) assays followed by Western blot analysis after co-overexpressing Flag-TRIM26 with GFP-p53 in p53-deficient H1299 cells (of note H1299 cells were used here because the transfection efficiency is very high). In line with these results, Flag-TRIM26 was co-immunoprecipitated with anti-GFP antibodies in the presence of GFP-p53, but not in the absence of GFP-p53 (Fig. 5f). This result was reproduced reciprocally with anti-Flag antibodies as shown in Fig. 5g. To further investigate the intracellular localization of TRIM26 interactions with p53 and MDM2, we performed immunoprecipitation assays using cell fractionation samples after overexpressing each protein (Fig. 5h, i). The results demonstrated that Flag-TRIM26 forms a complex with GFP-p53 in both the cytosol and nucleus (Fig. 5h), while it interacts with HA-MDM2 only in the cytosol (Fig. 5i). These results indicate that TRIM26 can interact with both p53 and MDM2.

To elucidate how TRIM26 facilitates the ubiquitination of p53 by MDM2, we examined the interaction between p53 and MDM2 in the absence or the presence of ecotopic TRIM26 using a Co-IP assay (Fig. 5j, k). The results indicated that the depletion of TRIM26 can weaken the endogenous p53-MDM2 interaction (Fig. 5j), while the overexpression of TRIM26 markedly enhanced the formation of the p53-MDM2 complex (Fig. 5k). This suggests that TRIM26 may form a complex with p53 and MDM2 to promote the effect of MDM2 on p53 protein ubiquitination.

### TRIM26 binds to C-termini of p53 and MDM2

To map their binding domains, we performed a set of GST-fusion protein-protein interaction assays by using purified GST-p53, GST-MDM2, and their GST-fragments proteins (lower panels of Fig. 6a, b). After incubating protein lysates containing TRIM26 with each of these GST-fusion proteins followed by intensive wash with binding buffers as described in the Materials and Methods, the bound TRIM26 was detected by Western blot analysis with anti-TRIM26 antibodies. As shown in Fig. 6a, c, TRIM26 bound to both the full length p53 and its C-terminal domain (aa291-393) that were fused with GST, but not to GST alone or other GST-p53 fragments. Interestingly, TRIM26 also bound to the full length MDM2 and its C-terminal domain (aa294-494) that were fused with GST, but not other the N- and central domains of MDM2 (Fig. 6b, d). Intriguingly, TRIM26 appeared to bind to the C-terminus of p53 more strongly than to the full length p53 (last two lanes of upper panel of Fig. 6a) even though the amounts of both the proteins used were equivalent (Fig. 6a, lower panel). This result suggests that the N-terminal and central domains of p53 might have a negative effect on the TRIM26 binding to the C-terminus of p53. This preference was not observed for TRIM26-MDM2 binding (last two lanes of upper panel of Fig. 6b). These results demonstrate that TRIM26 can bind to both p53 and MDM2 via their C-termini directly.

As shown in Fig. 6e–h, we also mapped the p53- and MDM2-binding domains of TRIM26 by performing GST-TRIM26 fusion protein-protein interaction assays with purified His-p53, His-MDM2, and GST-TRIM26 and GST-fragment proteins (Fig. 6e, f). The results showed that p53 binds to the C-terminal PRYSPRY domain (aa301-539) (Fig. 6e, g), whereas MDM2 binds to the N-terminal domain (aa1-300) containing the coiled-coil domain (Fig. 6f, h). MDM2 did not bind to other N-terminal fragments (aa1-150 and aa1-300) or the C-terminal domain. These findings indicate that p53 and MDM2 would not compete with each other when binding to TRIM26 as they bind to different domains of the latter.

## Discussion

TRIM26 is an under-studied E3 ubiquitin ligase, though it has been shown to play either an anti-cancer or oncogenic role in cancer development, depending on cancer types and its target proteins, over the past five years [4, 8]. Looking into the role of TRIM26 as a tumor suppressor, multiple studies support this effect, as TRIM26 can degrade and target the oncoproteins and pro-survival factors shows an anti-cancer role in various cancer types. Lu and Wu conducted research involving overexpressed TRIM26, which led to the downregulation of the pre-apoptosis gene p-AKT in endometrial cancer cells. This intervention resulted in a significant decrease in both tumor volume and weight within the endometrium [26]. Similarly, Tao et al. explored the same pathway of TRIM26’s impact, revealing its ability to suppress tumor growth in the setting of endometrial cancer by downregulating AKT phosphorylation [8]. Another study by Wang, Chai et al. found that TRIM26, acting as an inhibitor of the PI3K/Akt pathway, effectively suppressed Papillary Thyroid Carcinoma (PTC) [9]. Li et al. focused on Hepatocellular Carcinoma (HCC) influenced by TRIM26. This study uncovered that the E3 ubiquitin ligase activity of TRIM26 was previously shown to target ZEB1, an oncoprotein crucial for the development of HCC and thus to act as a tumor suppressor in HCC [12]. Additionally, another study conducted by Xia et al. has confirmed the anticancer behavior of TRIM26 through the downstream regulation of MEK/ERK. This leads to the inhibition of osteosarcoma proliferation [3]. Furthermore, in clear cell renal cell carcinoma (ccRCC) Shen, Wang et al. discovered that TRIM26 targets SNRBP, which directly participates in ubiquitination. This study reported a shorter lifespan in patients with low levels of TRIM26 [27].

Investigating its oncogenic propensity, Xie et al. uncovered that suppressing TRIM26 leads to the inhibition of proliferation, migration, and invasion of bladder cancer cells by impeding the AKT/GSK3β/β-catenin pathway [4]. A parallel effect was observed in glioblastoma as revealed by Mahlokozera et al. who found that TRIM26 stabilizes SOX2 protein and enhances its oncogenic activity in glioblastoma via its C-terminal PRYSPRY domain without engaging its Ring domain and E3 ligase activity [15]. Moreover, the oncogenic influence of TRIM26 was also detected in non-small cell lung cancer (NSCLC) by Sun et al. The study pointed out that TRIM26 serves as a ubiquitin ligase for PBX1, and depletion of TRIM26 hindered NSCLC growth [28].

Consistent with the TRIM26 oncogenic role described above, we report in this study that TRIM26 also plays an oncogenic role in CRC cells and possibly in melanoma cells by inactivating p53. First, overexpression of TRIM26 significantly enhanced the growth and proliferation of CRC HCT116 cells (Fig. 1) and the cells-derived xenograft tumors (Fig. 2). In line with these results, knockdown of TRIM26 reduced the proliferation and colony formation of these cells (Fig. 1). These outcomes appear to be p53-dependent, as the effect of either overexpression or knockdown of TRIM26 on HCT116 cell growth and proliferation was significantly impaired in the absence of p53 (Fig. 1). Indeed, overexpression of TRIM26 markedly reduced the level and activity of p53 in these cancer cells and the cells-derived xenograft tumor tissues (Figs. 2, 3 and S Fig. 3). The knockdown of TRIM26 led to increased p53 level and activity as represented by the rise of p21 protein and RNA level (S Fig. 3 and Fig. 3). Part of these results was repeated in melanoma cells (S Figs. 2 and 4). Hence, our findings reveal a new role for TRIM26 in negatively regulating p53 stability and activity in CRC cancer cells. By doing so, TRIM26 promotes the proliferation and growth of CRC cells and xenograft tumors derived from human CRC cells.

The E3 ubiquitin ligase activity of TRIM26 targeting ZEB1 and acting as a tumor suppressor in HCC was studied before [12], here we demonstrated that TRIM26 could induce the ubiquitination of the tumor suppressor p53, leading to its degradation (Fig. 4). TRIM26 can both initiate p53 ubiquitination independently and enhance MDM2-mediated p53 degradation. TRIM26 was able to initiate basal ubiquitination of p53 even in MEF-DKO cells (Fig. 4e). Notably, the ubiquitination of p53 increased significantly in the presence of MDM2 (Fig. 4e, f, S.Fig. 5a, b), suggesting that the interaction between p53 and MDM2 is strengthened when TRIM26 is overexpressed (Fig. 5k). Conversely, the interaction between p53 and MDM2 was suppressed when TRIM26 was depleted (Fig. 5j). Indeed, TRIM26 co-localized with both p53 and MDM2 in cells (Fig. 5a, b, S.Fig. 6d–f), and co-immunoprecipitation experiments in HCT116 cells confirmed the formation of an endogenous ternary complex of MDM2, p53, and TRIM26, immunoprecipitated with antibodies against TRIM26, p53, or MDM2 (Fig. 5c–e). Furthermore, protein-binding domain mapping revealed that TRIM26 binds to p53 independently of MDM2 and does not compete with MDM2 when ubiquitinating p53. This observation is reasonable because TRIM26 could bind to the C-terminal domains of both p53 (Fig. 6a, c) and MDM2 (Fig. 6b, d), while MDM2 can bind to both the C-terminal and the N-terminal domains of p53 with preference to the latter [29]. Additionally, p53 bound to the C-terminal domain of TRIM26 (Fig. 6e, g), in contrast, MDM2 (Fig. 6f, h) primarily bound to its N-terminal domain, further supporting that these two proteins do not competitively bind to TRIM26. Therefore, these results suggest that TRIM26 can bind to and ubiquitinate p53, leading to p53 destabilization either independently or in collaboration with MDM2. However, TRIM26 also has the potential to contribute to tumor growth by indirectly inactivating p53 through other targets. For example, Interferon Regulatory Factor 3 (IRF3), which acts as a tumor suppressor in colorectal cancer [30] and can activate p53 protein expression [31], has been reported to interact with TRIM26 in the nucleus, leading to its enhanced polyubiquitination and subsequent degradation [32].

In summary, our studies demonstrate that TRIM26 can act as an oncoprotein that promotes the proliferation and growth of CRC cells and xenograft tumors by destabilizing p53 via a ubiquitin-dependent mechanism (Fig. 6i). Our studies also raise a few outstanding questions. Does TRIM26 expression level affect CRC patients’ outcomes? Is it possible that TRIM26 can promote the growth and proliferation of other types of cancers that harbor wild-type p53, such as lung, bladder, melanoma (S Figs. 2, 4), or breast cancers? Is it possible that TRIM26 might act as a tumor suppressor by degrading mutant p53 in those malignant cancers that harbor mutated p53? Addressing these questions would help us depict a better picture of how TRIM26 acts as a dual player in tumorigenesis and as a potential drug target for future development of anti-cancer therapies.

## Materials and methods (See Supplementary Information for more)

### Plasmids and antibodies

The plasmids encoding Flag-TRIM26, Myc-His-TRIM26, or PLVX-Flag-TRIM26 were generated into the pCDNA3.1 vector, or PLVX vector. Plasmids encoding p53, Flag-p53, GFP-p53, HA-MDM2, His-Ub, HA-Ub, GST-p53 fragment, GST-MDM2 fragment, and GST-TRIM26 were previously described or generated using previously reported methods [33–36]. Anti-TRIM26 (Santa Cruz Biotechnology catalog no. K3018, 1:1000 dilution for IB and 1:100 for IF), Anti-TRIM26 (Proteintech catalog no.27013-1-AP, diluted 1:150 for immunofluorescence), anti-Flag (Sigma-Aldrich, catalog no. F1804, diluted 1:3,000 for IB), anti-HA (Proteintech catalog no.51064-2-AP, diluted 1:100 for immunofluorescence), anti-p53 (DO-1, Santa Cruz Biotechnology, catalog no. sc-126, diluted 1:1,000 for IB and 1:100 for immunofluorescence), anti-MDM2(Santa Cruz Biotechnology catalog no. A2921, diluted 1:1,000 for IB), anti-p21 (CP74, Neomarkers, Fremont, catalog no. MS-891-P0, diluted 1:1,000 for IB), anti-BAX (Santa Cruz Biotechnology catalog no. sc-493, diluted 1:100 for IB), anti-PARP (Proteintech catalog no.13371-1-AP, diluted 1:1,000 for IB), and anti-GAPDH (Proteintech, catalog no. 60004-1-Ig), diluted 1:10,000 for IB were commercially purchased. Antibodies against (2A9 and 4B11) were previously described [34, 35].

### Western blotting (WB)

As described previously [37], cells were harvested and lysed in lysis buffer consisting of 50 mM Tris/HCl (pH7.5), 0.5% Nonidet P-40 (NP-40), 1 mM EDTA, 150 mM NaCl, 1 mM dithiothreitol (DTT), 0.2 mM phenylmethylsulfonyl fluoride (PMSF), 10 mM pepstatin A and 1 mM leupeptin. Equal amounts of clear cell lysate (30–50 µg) were used for WB analyses.

### Reverse transcription and quantitative PCR analyses

Total RNA was isolated from cells using Trizol (Invitrogen, Carlsbad, CA, USA) following the manufacturer’s protocol. Total RNAs of 0.5 to 1 µg were used as templates for reverse transcription using poly-(T) 20 primers and M-MLV reverse transcriptase (Promega, Madison, WI, USA). Quantitative PCR (qPCR) was conducted using SYBR Green Mix according to the manufacturer’s protocol (BioRad, Hercules, CA, USA). The primers for human TRIM26 and p21 and P53 are as follows: TRIM26, F:5ʹ- GAACCACCTGAGTACCCTAAGG-3ʹ；R: 5ʹ-CTCAGCCACAATGTACTGCCTC-3ʹ. The primers for human p53, p21, were used as previously described [38, 39].

## Supplementary information

Tan and Ko et al Suppl Information

## Data Availability

All datasets are available from the corresponding authors on reasonable request.
