# Supplementary material for: Tripartite motif-containing protein 26 promotes colorectal cancer growth by inactivating p53
Source: Cell Death Differ. 2025 Feb 24;32(6):1142–52. doi: 10.1038/s41418-025-01463-1 (PMC12162846; doi:10.1038/s41418-025-01463-1)

SUPPLEMENTARY INFORMATION:

Tripartite motif-containing protein 26 promotes colorectal cancer growth by inactivating p53

Zhihui Tan[1,2,#], Hyun Min Ko[1,#], Parnian Naji[5], Rong Zhu[1,3], Jieqiong Wang[1], Shibo Huang[1,4], Yiwei Zhang[1], Shelya X. Zeng[1,*], and Hua Lu[1,*]

[1] Department of Biochemistry & Molecular Biology and Tulane Cancer Center, Tulane University School of Medicine, New Orleans, LA 70112, USA.

[2] Department of Gynecology, Xiang-Ya Hospital, Central South University, Changsha 410008, China.

[3] School of Life Sciences, Hubei University, Wuhan, Hubei province 430062, China;

[4] The Research Center for Clinical Trials, The First Affiliated Hospital, Nanchang University, Nanchang, Jiangxi 330006, China.

[5] Department of Surgery, Division of Surgical Oncology, University Hospitals Cleveland Medical Center, Cleveland, OH 44106, USA.

Running Title: TRIM26 Inactivation of p53

[#]: These authors have made equal contributions to the manuscript.

[*]Corresponding Author:

Hua Lu, Department of Biochemistry & Molecular Biology and Tulane Cancer Center, Tulane University School of Medicine, New Orleans, LA 70112, USA. Phone：504-988-0394; Email: hlu2@tulane.edu

Shelya X Zeng, Department of Biochemistry & Molecular Biology and Tulane Cancer Center, Tulane University School of Medicine, New Orleans, LA 70112, USA. Phone：504-988-3919; Email: szeng@tulane.edu

SUPPLEMENTARY MATERIALS AND METHODS

Cell culture and transient transfection

Human colorectal cancer, HCT116$^{(p53+/+)}$ and HCT116$^{(p53-/-)}$, lung cancer H1299, p53-null/MDM2-null murine embryonic fibroblast (MEF-DKO) cells, human melanoma SK-Mel5, and SK-Mel-147 cells were used in this study.

In our research endeavors, we were grateful to receive generous contributions from renowned scientists. Dr. Bert Vogelstein from the John Hopkins Medical Institutes kindly provided us with HCT116$^{p53+/+}$ and HCT116$^{p53-/-}$ cells. Additionally, we were fortunate to receive MEF-DKO cells as generous gifts from Dr. Guillermina Lozano at MD Anderson Cancer Center, the University of Texas. Human melanoma SK-Mel 5 and SK-Mel 147 cells were obtained from Dr. Shaomeng Wang at University of Michigan. Ensuring the integrity of our research, we conducted STR profiling to confirm the authenticity of the cell lines, while also confirming the absence of mycoplasma contamination.

All the cells were maintained in Dulbecco’s modified Eagle’s medium (DMEM) supplemented with 10% fetal bovine serum, 50 U/ml penicillin and 0.1 mg/ml streptomycin.

All cells were cultured at 37 ˚ C in a 5% CO 2 humidified atmosphere. Cells seeded on the plate overnight were transfected with plasmids using TurboFect transfection reagent following the manufacturer’s protocol (Thermo Scientific). Cells were harvested at 30-48 hours post-transfection for future experiments.

Generation of stable cell lines

HCT116$^{(p53+/+)}$ and HCT116$^{(p53-/-)}$ cells were transfected with PLVX-Flag-TRIM26 or the control vector PLVX-Flag-pCDNA. After 48 hours of transfection, the cells were cultured in selection medium containing 500 µg/ml of Geneticin 418. The selection medium was replaced every 3 days for the next 3 weeks. Subsequently, distinct colonies of surviving cells were transferred onto 6-well plates and the cultures were maintained under the same selection medium, and colonies with overexpression of PLVX-Flag-TRIM26 were detected by WB analysis using the

Flag antibody.

Cell proliferation assay

IncuCyte S3 Live-Cell Analysis System (Essen Bioscience) was used for kinetic monitoring of cell proliferation. Cells were plated into 96-well plates at a density of 2,000 - 3,000 cells per well for HCT116$^{(p53+/+)}$ and HCT$^{(p53-/-)}$. Cell proliferation was monitored using IncuCyte S3 live-cell imaging system every 3 h for 5 days. Data were analyzed by the IncuCyte S3 Basic Analyzer software module.

Colony formation assay

Cells were trypsinized and seeded at equal number of cells on 6-well plates. Media were changed every 3 days until the colonies were visible. Cells were fixed with methanol and stained with crystal violet solution at RT for 30 min. ImageJ was used for quantification of the colonies.

In vivo ubiquitination assay

HCT116$^{(p53-/-)}$, H1299, or MEF-DKO cells were transfected with plasmids encoding p53, Flag-p53, GFP-p53, HA-MDM2, His-Ub, HA-Ub or Flag-TRIM26 as indicated in the figure legends. At 42 hours after transfection, cells were treated with 20 μM MG132 for 6 h and then harvested and split into two aliquots, one for WB analysis and the other for ubiquitination assay. When His-Ub plasmid was used, cell pellets were lysed in buffer I (8 M urea, 0.1 M Na_2HPO_4 /NaH_2PO_4 (pH 8.0), 10 mM Tris-HCl (pH 8.0), 200 mM imidazole, 10 mM β-mercaptoethanol) and incubated with Ni-NTA beads (Qiagen) at RT for 4 hours. Beads were washed twice with buffer I and buffer II (8 M urea, 0.1 M Na_2HPO_4 /NaH_2PO_4 (pH 6.3), 10 mM Tris-HCl (pH 6.3), 10 mM β-mercaptoethanol). Proteins were eluted from beads in buffer III (200 mM imidazole, 0.15 M Tris-HCl (pH 6.7), 30% glycerol, 0.72 M β-mercaptoethanol, and 5% SDS). Eluted proteins were detected by WB analysis with indicated antibodies. H1299 cells and MEF-DKO

cells were used to determine the ubiquitination of endogenous p53 in the presence or absence of TRIM26 or MDM2 using the same assay as described above and also briefed in the legends for Figs. 4e-4f.

Cell fractionation

The cell fractionation assay was performed identically to the previous method (1). Briefly, cells were collected after transfection with each plasmid and washed with PBS. The cells were then resuspended in CE buffer (10 mM HEPES, 60 mM KCl, 1 mM EDTA, 0.075% NP-40, 1 mM DTT, and 1 mM PMSF, pH adjusted) and incubated on ice for 5 minutes. The cytoplasmic extract was collected by centrifugation. The nuclei pellet was then washed with CE buffer without NP-40. The nuclei pellet was subsequently resuspended in NE buffer (20 mM Tris/HCl, 420 mM NaCl, 1.5 mM MgCl2, 0.2 mM EDTA, 1 mM PMSF, and 25% glycerol, adjusted to pH 8.0) and periodically incubated on ice for 10 minutes. Finally, the cytoplasmic and nuclear extracts were separated by centrifugation at 13,000 RPM for 10 minutes.

Immunofluorescence (IF) staining

Cells were fixed with 4% paraformaldehyde for 15 minutes and then permeabilized with 0.2% Triton X-100 for 15 minutes. Next, the cells were blocked with 1% BSA solution at RT for 30 minutes. They were then incubated overnight at 4°C with primary antibodies. The following day, the cells were incubated with secondary antibodies (Alexa Fluor 488 or Alexa Fluor 594) (Invitrogen-Thermo, CA) at RT for 2 hours. For co-staining, additional primary antibodies were incubated at RT for 2 hours, followed by incubation with secondary antibodies for another 2 hours. Finally, DAPI (Sigma) staining was performed to distinguish the nuclei. Images of the stained cells were acquired using confocal microscopy (Nikon TiE-2, Nikon Inc., Tokyo, Japan).

Immunoprecipitation (IP)

IP was conducted using antibodies as indicated in the figure legends. After the cells were

collected and lysed with lysis buffer (50 mM Tris/HCl (pH7.5), 0.5% Nonidet P-40 (NP-40), 1 mM EDTA, 150 mM NaCl, 1 mM dithiothreitol (DTT), 0.2 mM phenylmethylsulfonyl fluoride (PMSF), 10 mM pepstatin A and 1 mM leupeptin). Briefly, 0.5-2 mg of proteins were incubated with the indicated antibody at 4℃ for 4h or overnight. Protein A or G beads (Santa Cruz Biotechnology) were then added, and the mixture was incubated at 4℃ for an additional 2 hours. Beads were washed at least three times with lysis buffer. Bound proteins were detected by IB with antibodies as indicated in the figure legends.

GST fusion protein association assay

GST-tagged MDM2 fragments, p53 fragments, or TRIM26 fragments were expressed in E. coli and conjugated with glutathione-Sepharose 4B beads (Sigma-Aldrich). Protein-protein interaction assays were conducted using cell lysates with Flag-TRIM26 or His-purified p53 or MDM2. Briefly, the cell lysates with Flag-TRIM26 or His-purified p53 or MDM2 were incubated and gently rotated with the glutathione-Sepharose 4B beads containing 500 ng of GST-MDM2 fragments, GST-p53 fragments, GST-TRIM26 fragments or GST only at RT for 40 min. The mixtures were washed three times with GST lysis buffer (50 mM Tris/HCl pH 8.0, 0.5% NP-40, 1 mM EDTA, 150 mM NaCl, 10% glycerol). Bound proteins were analyzed by WB with the antibodies as indicated in the figure legends.

Mouse xenograft experiments

Five-week-old female J:NU/homozygous nude (*Foxn1^{nu}/Foxn1^{nu}*) mice were obtained from The Jackson Laboratory (Bar Harbor, ME, USA). Thirty-two nude mice were divided randomly into two groups for tumor xenografts as HCT116$^{(p53+/+)}$ and HCT$^{(p53-/-)}$ group. Stable control and TRIM26 overexpressed HCT116$^{(p53+/+)}$ and HCT116$^{(p53-/-)}$ cells were generated as described above. The 1 x 10^6 control cells or TRIM26 overexpressed cells were injected into the flanks of each mouse, at two (left for control and right for overexpression) sides. The tumor size and the mice weights were monitored and recorded every 2 days, and the tumor volume was

calculated as mm³ = length x (width)² x 0.5 and presented in a graph (Fig. 2c-2d). When the biggest tumor grew up to approximately 1 cm³, mice were euthanized. The tumors were harvested, and tumor weights were measured and presented in histograms. This animal study received approval from the Institutional Animal Care and Use Committee at Tulane University School of Medicine.

Statistics

All in vitro experiments were performed at least 2 or more replicates. The student's two-tailed t-test and one-way analysis of variance (ANOVA) were used to determine mean difference among groups. $P < 0.05$ was considered statistically significant. Data are presented as mean ± s.e.m.

REFERENCES

1. Li C, Lee H. Coiled-coil domain containing 3 suppresses breast cancer growth by protecting p53 from proteasome-mediated degradation. 2023;42(2):154-64.

SUPPLEMENTARY FIGURE LEGENDS

S Figure 1. TRIM26 is overexpressed in multiple cancers. The pan-cancer analysis of TRIM26 was conducted based on the bioinformatic data downloaded from TNMplot (https://tnmplot.com/analysis/). Red means significant differences by Mann-Whitney U test $p<0.05$. The table shows the number of samples that were reorganized in Excel.

S Figure 2. TRIM26 promotes colony formation of skin melanoma cells.

(**a, b**) TRIM26 promotes colony formation. SK-Mel-5 or SK-Mel-147 cells that harbor wild type p53 were transfected with Myc-His-TRIM26 or Myc-His-control, Si-TRIM26, Si-control, in SK-Mel-5 and SK-Mel-147 and seeded in 6-well plates for 14 days: (**a**): Cell colony formation after overexpression of TRIM26. (**b**): Cell colony formation after knockdown of TRIM26. Histograms indicate the relative colony number for cells with (**c**) overexpression of TRIM26, or with (**d**) knockdown of TRIM26 (*$P < 0.05$, ***$P < 0.001$).

S Figure 3. TRIM26 suppresses p53 level and activity in human colon cancer cells.

(**a, b**): Protein levels after (**a**) knocking down or (**b**) overexpression TRIM26 in HCT116$^{(p53+/+)}$ cells. The cells were transfected with Si-TRIM26, Si-control, Myc-His-TRIM26 or Myc-His-control for 48 h and harvested for WB analysis with indicated antibodies.

S Figure 4. TRIM26 suppresses p53 level and activity in human melanoma cells.

(**a, b**): Protein levels after (**a**) knocking down or (**b**) overexpression TRIM26 in SK-Mel-5 or SK-Mel-147 cells. The cells were transfected with Si- TRIM26, Si-control, Myc-His-TRIM26 or Myc-His-control for 48 h and harvested for WB analysis with indicated antibodies.

S Figure 5. Quantitative analysis of p53 ubiquitination performed in the experiments shown in Figures 4e and 4f.

(**a, b**) Quantification of ubiquitinated p53 in (**a**) MEF-DKO cells and (**b**) H1299 cells. Quantification of ubiquitinated p53 was performed using image J software. Statistical analysis was performed by one-way ANOVA. Data are represented as mean ± s.e.m. *P<0.05, ***P < 0.001, ****P < 0.0001 vs. lane 2. **P < 0.01 vs lane 3.

S Figure 6. Localization patterns of TRIM26, p53, or MDM2 overexpressed alone or together were detected by immunofluorescence assay in HCT116$^{(p53+/+)}$.

(**a-c**) Localization of exogenous (**a**) TRIM26, (**b**) p53, or (**c**) MDM2 in CRC cells. Cells were transfected with Flag-TRIM26, GFP-p53, or HA-MDM2 alone for 48 hours. After transfection, cells were fixed, permeabilized, blocked, and stained with specific tag antibodies, followed by secondary antibody staining. DAPI was used to visualize the nucleus. (Scale bar, 10 μm). (**d-f**) Colocalization patterns of exogenous TRIM26 with (**d, e**) exogenous p53 in the nucleus and with (**f**) exogenous MDM2 in the cytoplasm in CRC cells. Cells were co-transfected with Myc-His-TRIM26 or Flag-TRIM26 along with GFP-p53 or HA-MDM2 for 48 hours. After transfection, cells were fixed, permeabilized, blocked, and stained with specific tag antibodies, followed by secondary antibody staining. DAPI was used to visualize the nucleus. (Scale bar, 20 μm).

Supplementary Figure 1

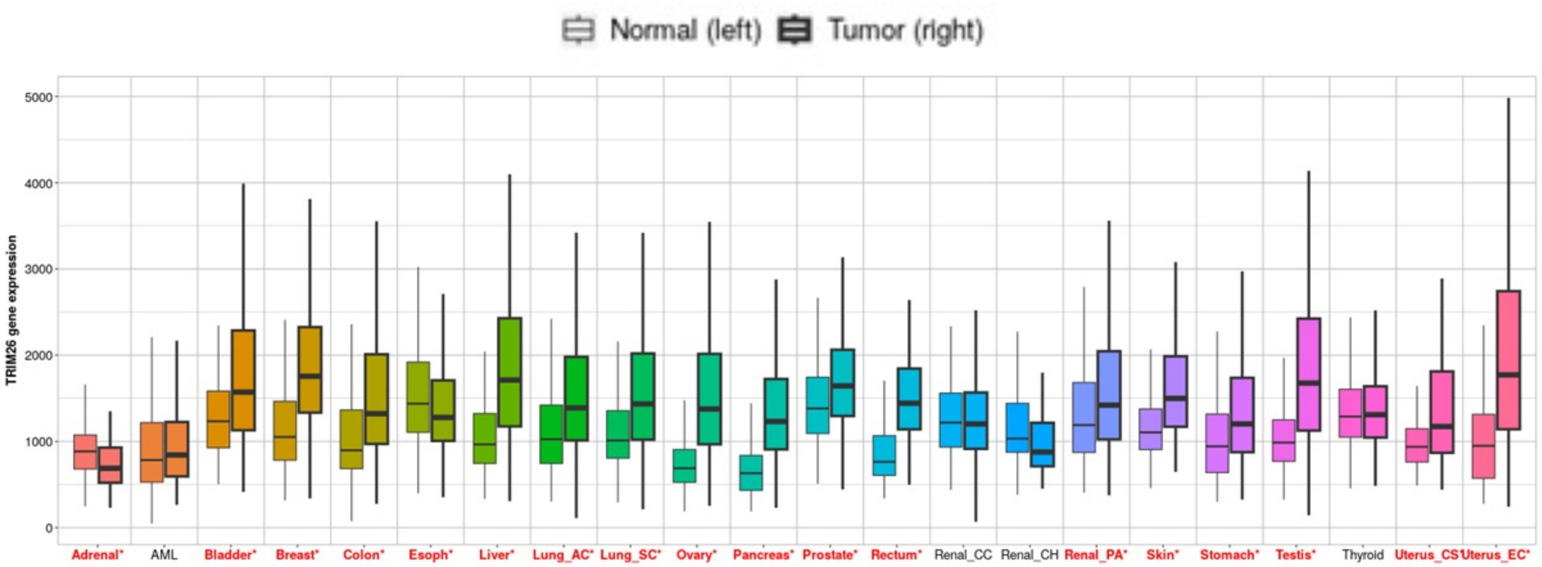

	Adrenal	AML	Bladder	Breast	Colon	Esoph	Liver	Lung_AC	Lung_SC	Ovary	Pancreas	Prostate	Rectum	Renal_CC	Renal_CH	Renal_PA	Skin	Stomach	Testis	Thyroid	Uterus_CS	Uterus_EC
N:	190	407	30	403	274	418	225	486	476	133	252	204	243	117	69	77	474	294	259	504	111	146
T:	79	151	411	1097	469	161	371	524	501	374	177	498	166	535	65	289	103	375	156	502	56	547

Supplementary Figure 2

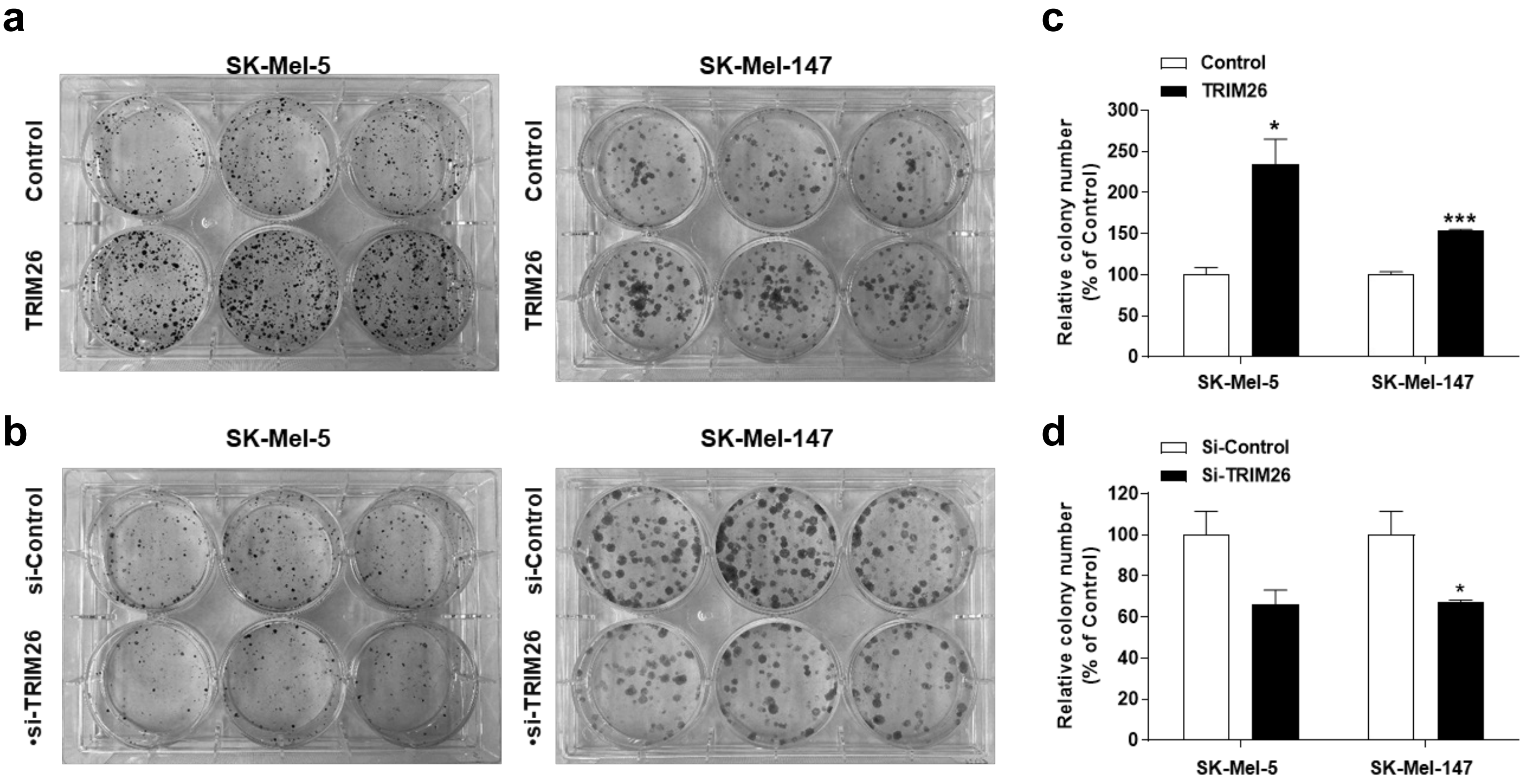

Supplementary Figure 3

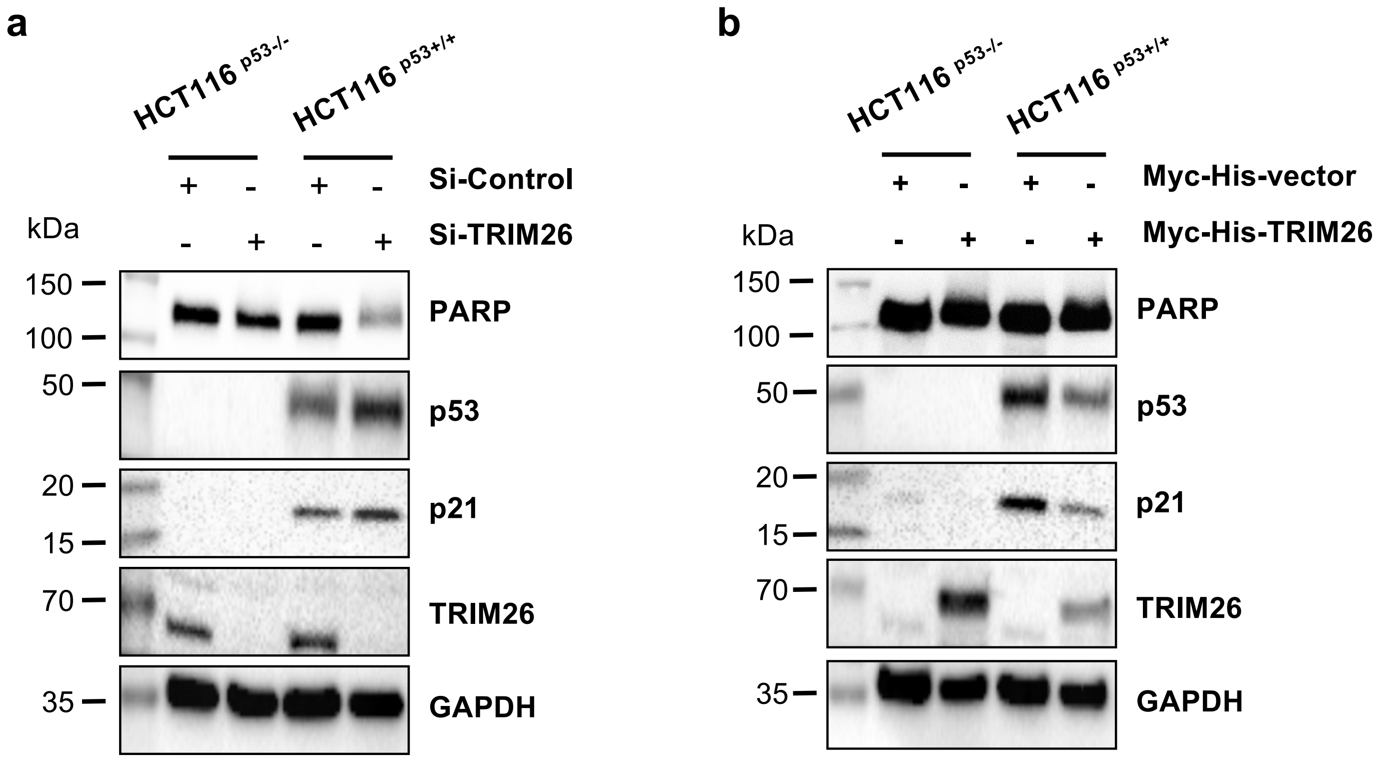

Supplementary Figure 4

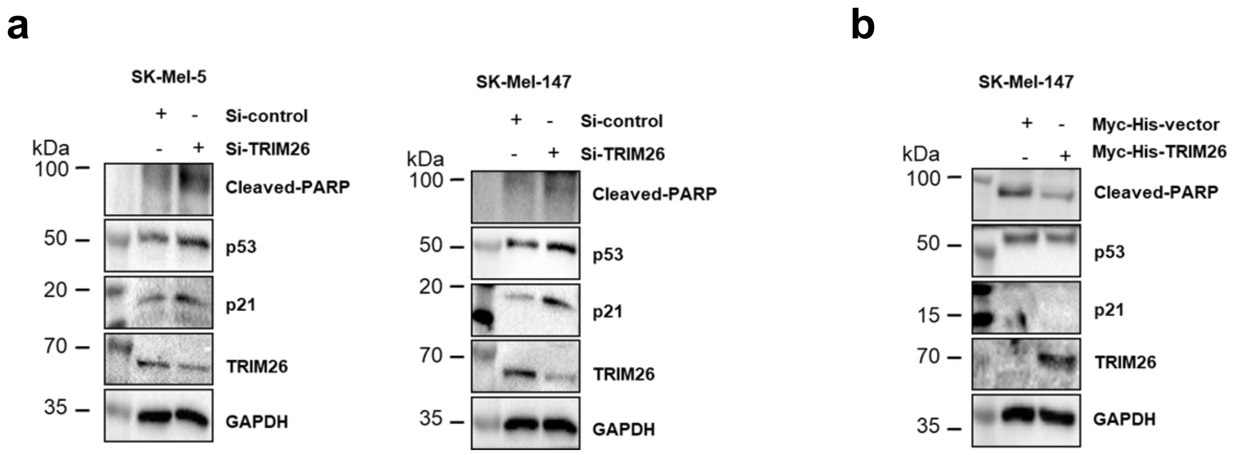

Supplementary Figure 5

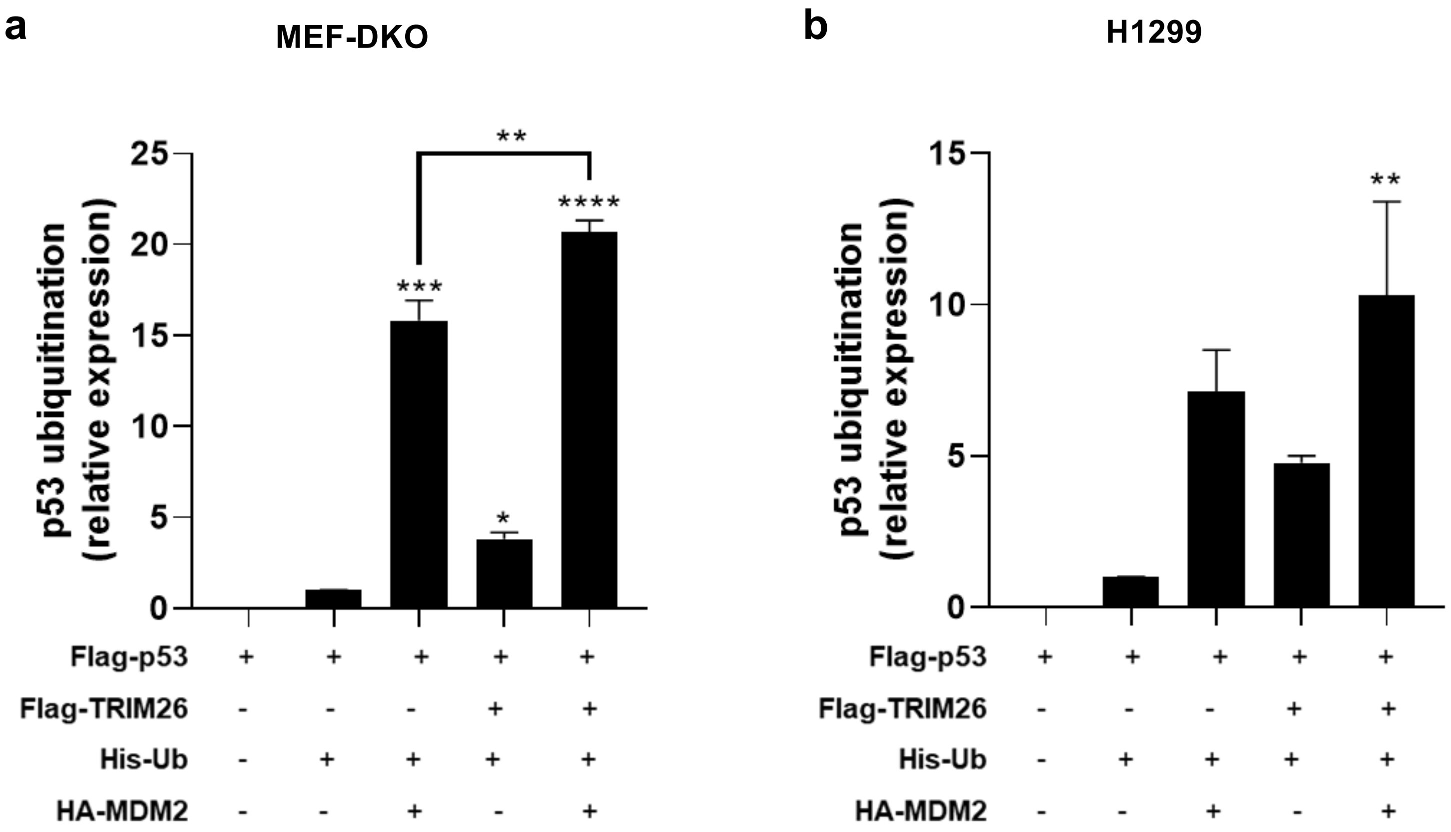

Supplementary Figure 6

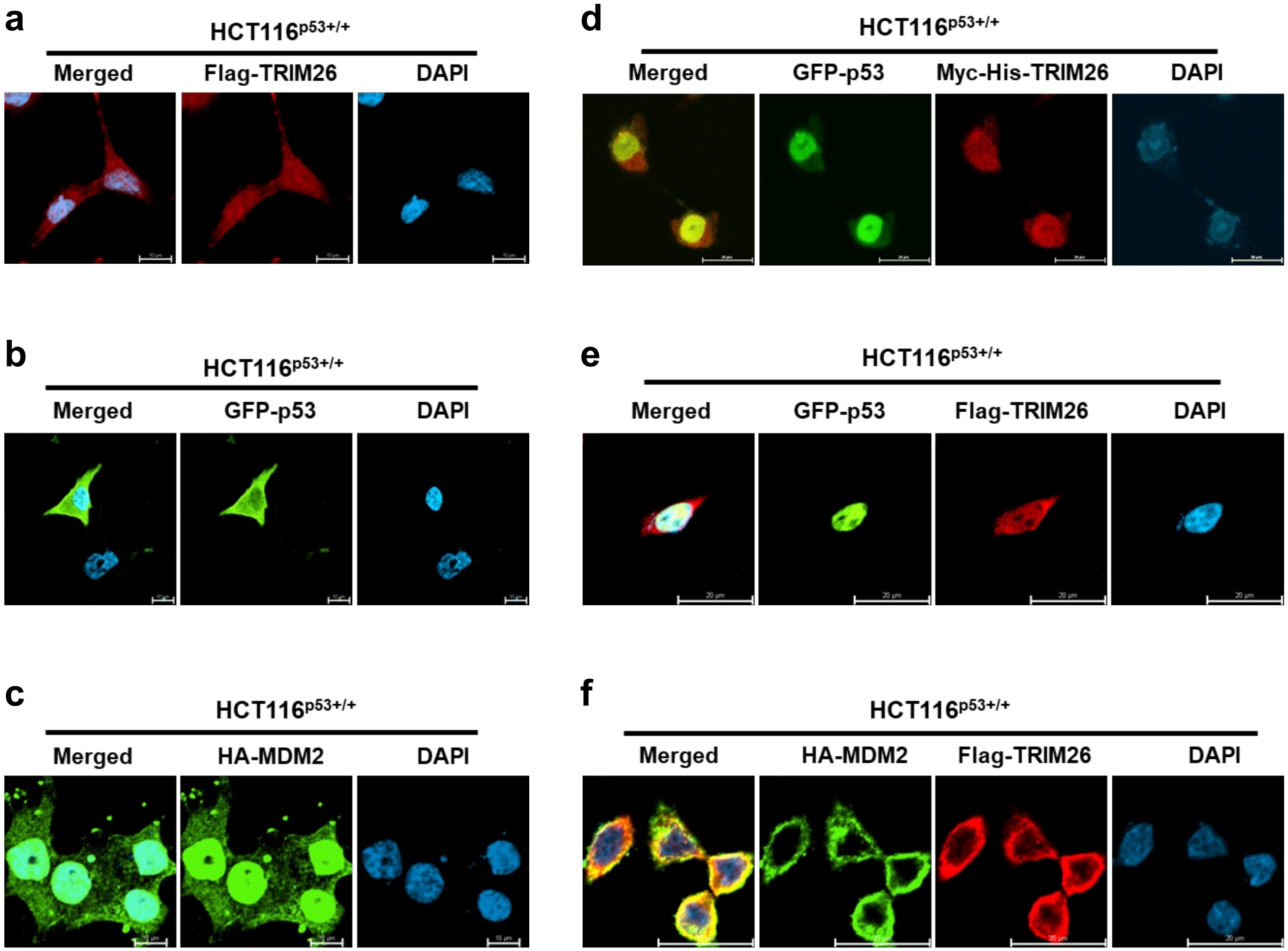

Supplement: Supplementary file 1 — Tan and Ko et al Suppl Information [file 41418_2025_1463_MOESM1_ESM.pdf]
